# Intraduodenal Administration of L-Valine Has No Effect on Antropyloroduodenal Pressures, Plasma Cholecystokinin Concentrations or Energy Intake in Healthy, Lean Men

**DOI:** 10.3390/nu11010099

**Published:** 2019-01-05

**Authors:** Rachel A. Elovaris, Penelope C. E. Fitzgerald, Vida Bitarafan, Sina S. Ullrich, Michael Horowitz, Christine Feinle-Bisset

**Affiliations:** Adelaide Medical School and National Health and Medical Research Council of Australia Centre of Research Excellence in Translating Nutritional Science to Good Health, Level 5 Adelaide Health and Medical Sciences Building, Corner North Terrace and George Street, Adelaide 5005, Australia; rachel.elovaris@adelaide.edu.au (R.A.E.); penelope.fitzgerald@adelaide.edu.au (P.C.E.F.); vida.bitarafan@adelaide.edu.au (V.B.); sina.ullrich@uniklinik-freiburg.de (S.S.U.); michael.horowitz@adelaide.edu.au (M.H.)

**Keywords:** branched-chain amino acids, gut motility, gut hormones, appetite regulation, glycaemia, human

## Abstract

Whey protein is rich in the branched-chain amino acids, L-leucine, L-isoleucine and L-valine. Thus, branched-chain amino acids may, at least in part, mediate the effects of whey to reduce energy intake and/or blood glucose. Notably, 10 g of either L-leucine or L-isoleucine, administered intragastrically before a mixed-nutrient drink, lowered postprandial blood glucose, and intraduodenal infusion of L-leucine (at a rate of 0.45 kcal/min, total: 9.9 g) lowered fasting blood glucose and reduced energy intake from a subsequent meal. Whether L-valine affects energy intake, and the gastrointestinal functions involved in the regulation of energy intake, as well as blood glucose, in humans, is currently unknown. We investigated the effects of intraduodenally administered L-valine on antropyloroduodenal pressures, plasma cholecystokinin, blood glucose and energy intake. Twelve healthy lean men (age: 29 ± 2 years, BMI: 22.5 ± 0.7 kg/m^2^) were studied on 3 separate occasions in randomised, double-blind order. Antropyloroduodenal pressures, plasma cholecystokinin, blood glucose, appetite perceptions and gastrointestinal symptoms were measured during 90-min intraduodenal infusions of L-valine at 0.15 kcal/min (total: 3.3 g) or 0.45 kcal/min (total: 9.9 g), or 0.9% saline (control). Energy intake from a buffet-meal immediately after the infusions was quantified. L-valine did not affect antral, pyloric (mean number; control: 14 ± 5; L-Val-0.15: 21 ± 9; L-Val-0.45: 11 ± 4), or duodenal pressures, plasma cholecystokinin (mean concentration, pmol/L; control: 3.1 ± 0.3; L-Val-0.15: 3.2 ± 0.3; L-Val-0.45: 3.0 ± 0.3), blood glucose, appetite perceptions, symptoms or energy intake (kcal; control: 1040 ± 73; L-Val-0.15: 1040 ± 81; L-Val-0.45: 1056 ± 100), at either load (*p* > 0.05 for all). In conclusion, intraduodenal infusion of L-valine, at loads that are moderately (3.3 g) or substantially (9.9 g) above World Health Organization valine requirement recommendations, does not appear to have energy intake- or blood glucose-lowering effects.

## 1. Introduction

High-protein diets, when compared with diets high in carbohydrate or fat, appear to have more potent effects to decrease appetite and body weight and improve glycaemic control in obese people with and without type 2 diabetes [1,2,3,4]. Whey protein appears to be particularly effective and has been shown to decrease energy intake as well as the blood glucose response to a meal [5,6], associated with changes in gastrointestinal (GI) motor and hormone functions [7,8,9,10]. For example, administration of whey directly into the duodenum (designed to avoid the potentially confounding effects of orosensory influences and differences in gastric emptying), at rates of 0.5–3 kcal/min over 60 min (equivalent to ~8–48 g protein), stimulated pyloric pressures, a key determinant of the slowing of gastric emptying, and the release of the gut hormones, cholecystokinin (CCK) and glucagon-like peptide-1 (GLP-1), in a dose-dependent fashion, and, at the highest rate, also suppressed energy intake [9]. In people with type 2 diabetes, managed by diet, ingestion of 55 g whey protein, 30 min before a carbohydrate meal, was shown to stimulate insulin, GLP-1 and glucose-dependent insulinotropic peptide (GIP) release and slow gastric emptying, associated with a marked reduction in postprandial glucose [11]. These effects were more marked than when the same amount of whey was co-ingested with the carbohydrate meal [11].

There is increasing evidence that, in analogy to triglycerides and fatty acids [12,13], amino acids may mediate the effects of protein, including whey, on appetite and glycaemia. Whey protein is rich in the branched-chain amino acids (BCAAs), L-leucine, L-isoleucine and L-valine. In response to intraduodenal administration of whey protein, we have observed direct correlations between plasma concentrations of these amino acids with plasma concentrations of GLP-1 and insulin, and an inverse correlation with energy intake [14], supporting the concept that these amino acids, at least in part, mediate the effects of whey to reduce energy intake and/or blood glucose. Indeed, both L-leucine and L-isoleucine have been shown in studies by ourselves and others to modulate gut motor and hormone functions, energy intake and/or blood glucose [15,16,17,18,19]; however, there is very little information about the effects of L-valine.

There is evidence that L-leucine decreases energy intake in both animals and humans [15,16]. In rats, acute injection of L-leucine, directly into the brain, inhibited food intake apparently by stimulating L-leucine-sensitive neurons in the nucleus of the solitary tract, suggesting central effects of L-leucine in the regulation of energy intake [16]. In humans, we have reported that intraduodenal infusion of L-leucine, at a rate of 0.45 kcal/min for 90 min, stimulated plasma CCK and reduced energy intake from a subsequent meal by a substantial 156 ± 57 kcal [15]. Both L-leucine and L-isoleucine also decrease fasting and postprandial blood glucose. In the study referred to above [15], intraduodenal L-leucine stimulated insulin and reduced blood glucose, in the absence of carbohydrate. Moreover, 10 g L-leucine or L-isoleucine, ingested orally with 25 g of glucose [17,18], or administered intragastrically 15 min before a carbohydrate-containing mixed-nutrient drink [19], attenuated the postprandial rise in blood glucose significantly.

The outcomes of studies conducted by ourselves [19,20] and others [21,22] indicate that the effects (and mechanisms) of amino acids to decrease appetite and glycaemia vary substantially. In fasted healthy humans, intravenous infusion of 30 g L-valine slightly decreased blood glucose and stimulated insulin [21]. Whether L-valine, when given at doses that are modestly (3.3 g) or substantially (9.9 g) above World Health Organization requirement recommendations for L-valine (~26 mg/kg body weight, equivalent to ~2 g for a 70 kg person [23]), but comparable to doses that have previously been shown to be effective for L-leucine and L-isoleucine [17,18,19], affects energy intake, or the gut functions associated with the regulation of energy intake or blood glucose, is currently unknown.

The aim of the present study was to evaluate the hypothesis that intraduodenal infusion of L-valine would modulate plasma hormone concentrations and upper GI motility, in a dose-dependent manner, associated with reduced energy intake and fasting glucose in healthy males.

## 2. Materials and Methods

### 2.1. Participants

Twelve healthy males, aged 29 ± 2 years (range: 22–43 years) and of normal body weight (body mass index 22.5 ± 0.7 kg/m^2^, range: 20–25 kg/m^2^) were recruited from an existing pool of volunteers and by flyers placed around the Royal Adelaide Hospital, University of Adelaide, University of South Australia and Flinders University campuses, and through advertisements in local newspapers and on online sites (University of Adelaide and Gumtree). Subjects who smoked, consumed >20 g of alcohol/day, had low ferritin (<30 ug/L) or iron (<8 umol/L) concentrations, were lactose-intolerant, vegetarians, or high-performance athletes, had significant GI symptoms, disease or surgery, or used medications known to affect GI functions and/or appetite, were excluded. All subjects were unrestrained eaters (score ≤12 on the eating restraint component of the Three-Factor Eating Questionnaire [24]) and had been weight-stable (<5% fluctuation) in the 3 months preceding the study. The study protocol was approved by the Human Research Ethics Committee of the Central Adelaide Local Health Network, and the study performed in accordance with the Declaration of Helsinki and the NHMRC Statement on Ethical Conduct in Human Research. Each subject provided written, informed consent prior to their enrolment. The study was registered as a clinical trial with the Australian New Zealand Clinical Trials Registry (www.anctr.org.au, ACTRN12617000715370).

### 2.2. Study Design

The study evaluated the dose-related effects of 90-min intraduodenal infusions of L-valine at loads of (i) 0.15 kcal/min (3.3 g over 90 min, “L-Val-0.15”) or (ii) 0.45 kcal/min (9.9 g over 90 min, “L-Val-0.45”), or (iii) saline (“control”), on antropyloroduodenal pressures, plasma CCK and blood glucose concentrations, appetite perceptions and energy intake in healthy male subjects.

### 2.3. Intraduodenal Infusions

L-valine solutions were prepared by dissolving 4.6 g or 13.8 g crystalline L-valine (PureBulk, Roseburg, Oregon, USA), NaCl (2.2 g and 0.8 g, respectively) and 146 mg CaCl_2_ × 2H_2_O, in distilled water to a final volume of 500 mL. The control solution contained 4.8 g NaCl and 146 mg CaCl_2_ × 2H_2_O dissolved in distilled water to a volume of 500 mL. All solutions were isotonic (300 mosmol) and had a pH of 7. Intraduodenal infusions were delivered at a rate of 4 mL/min, thus, delivering loads of L-valine at 0.15 kcal/min (total: 3.3 g) or 0.45 kcal/min (total: 9.9 g), in a total volume over 90 min of 360 mL. The loads were based on our previous study, which found significant effects of intraduodenal L-leucine on upper GI functions, blood glucose and energy intake [15]. Solutions were prepared on the morning of each study day, and were administered in a randomised, double-blind fashion. Both the preparation of the solutions and the randomisation (using an online tool [25]) were performed by a research officer, who was not involved in the performance of the studies or data analysis.

### 2.4. Study Protocol

Each subject was studied on three occasions, separated by 3–7 days. Subjects were instructed to abstain from vigorous exercise and alcohol intake for 24 h before each study visit. They were provided with a standardised evening meal (Beef Lasagne; McCain Food; energy content: 602 kcal) to be consumed by 7:00 p.m. on the night before each study, after which time they fasted overnight from solids and liquids. Subjects then attended the Clinical Research Facility at the University of Adelaide at 8:00 a.m. the following morning. They were intubated with a small-diameter (external diameter: 3.5 mm), 17-channel manometric catheter (length: 100 cm; Dentsleeve International, Mui Scientific, Mississauga, ON, Canada), which was inserted into the stomach through an anaesthetised nostril and allowed to pass through the pylorus into the duodenum by peristalsis [8,26]. The manometric catheter contained 16 side-holes spaced at 1.5 cm intervals and was positioned with six side-holes in the antrum (channels 1–6), a 4.5-cm sleeve sensor (channel 7), with two side-holes (channels 8, 9) on the back of the sleeve, across the pylorus, and seven side-holes in the duodenum (channels 10–16). The correct positioning of the catheter, with the sleeve sensor straddling the pylorus, was maintained by continuous measurement of the transmucosal potential difference between the most distal antral, and the most proximal duodenal, channels [8,27]. An additional channel (with the side-hole positioned ~14.5 cm distal to the pylorus when the catheter was in position) was used for intraduodenal infusion of L-valine or control solutions.

Once the catheter was positioned correctly, fasting motility was observed until the occurrence of a phase III of the migrating motor complex. Immediately after phase III passed the antropyloroduodenal region, and during a subsequent period of motor quiescence (phase I), an intravenous cannula was placed in a forearm vein for blood sampling. At *t* = −10 min (approximately 10:30 a.m.) and *t* = 0 min, fasting blood samples were taken, and the subject completed visual analogue scale (VAS) questionnaires to assess appetite perceptions and GI symptoms. The intraduodenal infusion of L-valine or control was then commenced and continued for 90 min (*t* = 0–90 min). Antropyloroduodenal motility was recorded continuously during the pre-infusion period and throughout the infusion, and blood samples for measurements of plasma CCK and blood glucose concentrations were collected, and VAS questionnaires completed, at 15-min intervals. At *t* = 90 min, the infusion was terminated and the catheter removed. The subject was then presented with a standardised, cold, buffet-style test meal [28]. Subjects were instructed to consume as much food as they wished until they felt comfortably full over up to 30 min (*t* = 90–120 min). The meal consisted of 4 slices (~120 g) each of whole-meal and white breads, 100 g sliced ham, 100 g sliced chicken, 85 g sliced cheddar cheese, 100 g sliced tomato, 100 g sliced cucumber, 100 g iceberg lettuce, 20 g margarine, 22 g mayonnaise, 120 g fruit salad, 175 g strawberry yoghurt, 100 g chocolate custard, 1 apple (~170 g), 1 banana (~190 g), 600 mL water, 350 mL orange juice and 375 mL iced coffee total energy content: ~2300 kcal; total weight: ~2924 g; macronutrient distribution: (~27% fat, ~52% carbohydrate and ~21% protein). Immediately after the meal (i.e., at *t* = 120 min), the intravenous catheter was removed, and the subject was then allowed to leave the laboratory.

### 2.5. Measurements

#### 2.5.1. Energy Intake

Each food item in the buffet meal was weighed before and after consumption to quantify the amount of food eaten (g). Energy intake (kcal) was then calculated using commercially available software (Foodworks 8.0, Xyris Software, Highgate Hill, Brisbane, QLD, Australia).

#### 2.5.2. Appetite Perceptions and GI Symptoms

Perceptions of appetite (hunger, fullness, desire to eat and prospective food consumption) were assessed using validated 100-mm VAS questionnaires [29]. GI symptoms (nausea and bloating) were also assessed. VAS scales consisted of 100-mm horizontal lines, where 0 mm represented “not felt at all”, and 100 mm “felt the strongest possible”. Subjects were asked to place a vertical mark on each horizontal line to rate the strength of each sensation felt at that point in time.

#### 2.5.3. Antropyloroduodenal Pressures

Antropyloroduodenal pressures were digitised and recorded via a computer-based system running commercially available software (MMS Database software, version 8.17; Solar GI, Enschede, The Netherlands). Antropyloroduodenal pressures were analysed for: (i) numbers and amplitudes of antral and duodenal pressure waves (PWs); (ii) numbers and amplitudes of isolated pyloric pressure waves (IPPWs); and (iii) basal pyloric pressure. Pressure waves were defined by an amplitude ≥10 mmHg, and a minimum interval of 15 s between peaks for antral and pyloric waves and 3 s for duodenal waves. Basal pyloric pressure was calculated by subtracting the mean basal pressure (with the exclusion of phasic pressures) recorded at the most distal antral side hole from the mean basal pressure recorded at the sleeve [27]. This analysis was performed using custom-written software (by Prof. A. Smout, University Medical Centre, Amsterdam, The Netherlands).

#### 2.5.4. Plasma CCK and Blood Glucose Concentrations

Blood samples were collected into ice-chilled ethylenediaminetetraacetic acid-coated tubes and centrifuged (3200 rpm at 4 °C for 15 min) within 15 min of collection to obtain plasma. Plasma samples were stored at −80 °C until analysed. Plasma cholecystokinin-8 (CCK-8) concentrations (pmol/L) were analysed by radioimmunoassay after ethanol extraction using an adaption of the method by Santangelo et al. [30]. The antibody used recognises sulfated CCK-8 and does not bind to structurally unrelated peptides. Cross-reactivity with unsulfated CCK-8 was ~15% and with human gastrin I 0.2%. The detection limit was 1 pmol/L, and intra-assay and inter-assay coefficients of variation were 9.2% and 13.7%, respectively.

Blood glucose concentrations (mmol/L) were determined immediately after collection by the glucose oxidase method using a portable glucometer (FreeStyle OptimumH; Abbott Laboratories, Chicago, IL, USA).

### 2.6. Data and Statistical Analyses

The number of subjects was based on power calculations derived from previous work [15]. We calculated that *n* = 12 subjects would be required to detect a 15% decrease in energy intake at α = 0.05, with a power of 80%.

For all data baseline values were calculated as means of values acquired between *t* = −10 and *t* = 0 min. During the 90-min infusion period, VAS scores, plasma CCK and blood glucose data were expressed as means at each time point, while the number of antral, isolated pyloric and duodenal PWs were expressed as total numbers, and basal pyloric pressures and the amplitude of antral, isolated pyloric and duodenal PWs, were expressed as mean values over the 90-min period. The numbers and amplitudes of antral and duodenal PWs were used to calculate antral and duodenal motility indices (MIs), as previously described [31]. 

Statistical analysis was performed with the use of SPSS software (version 24; IBM, Armonk, NY, USA). VAS scores, plasma CCK and blood glucose data were analysed using repeated-measures two-factor analysis of variance (ANOVA), with time (0–90 min), and treatment (L-valine-0.15, L-valine-0.45, control) as factors. MIs for antral and duodenal PWs, basal pyloric pressure, number and amplitude of IPPWs and energy intake were analysed using one-factor ANOVA. Sphericity of the time effect for all models was evaluated by Mauchly’s test, and, if violated, the adjusted Greenhouse-Geisser *p* value was reported. Post-hoc comparisons, adjusted for multiple comparisons by Bonferroni correction, were performed where ANOVAs revealed significant effects. All data are reported as means ± SEMs. All tests were two-tailed and statistical significance was accepted at *p* < 0.05.

## 3. Results

All subjects completed the 3 study visits and tolerated the study treatments well.

### 3.1. Energy Intake

**Figure 1:**
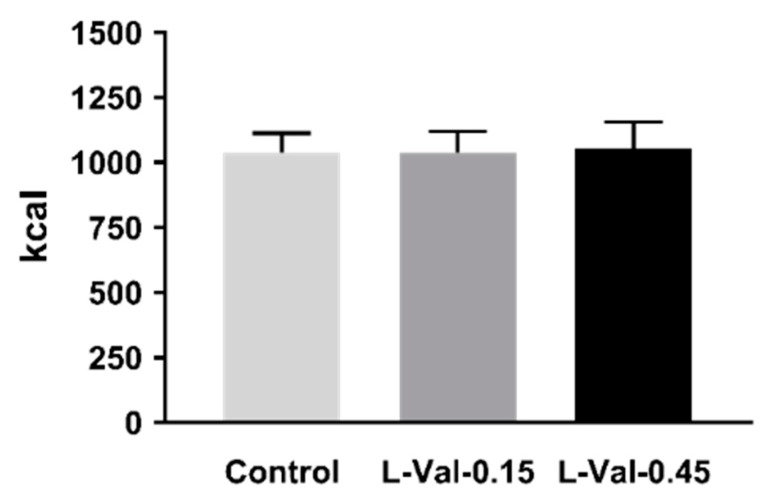
Energy intake from a buffet meal after 90-min intraduodenal infusions of control, or L-valine at 0.15 kcal/min (“L-Val-0.15”) or 0.45 kcal/min (“L-Val-0.45”). One-way ANOVA was used to analyse the data. Statistical significance was accepted at *p* < 0.05. Data are means ± SEMs, *n* = 12.

There was no effect of treatment on energy intake (Figure 1), or the amount eaten (gram; control: 1198 ± 94; L-Val-0.15: 1156 ± 83; L-Val-0.45: 1156 ± 97), from the buffet-meal (*p* > 0.05 for both).

### 3.2. Appetite Perceptions and GI Symptoms

**Figure 2:**
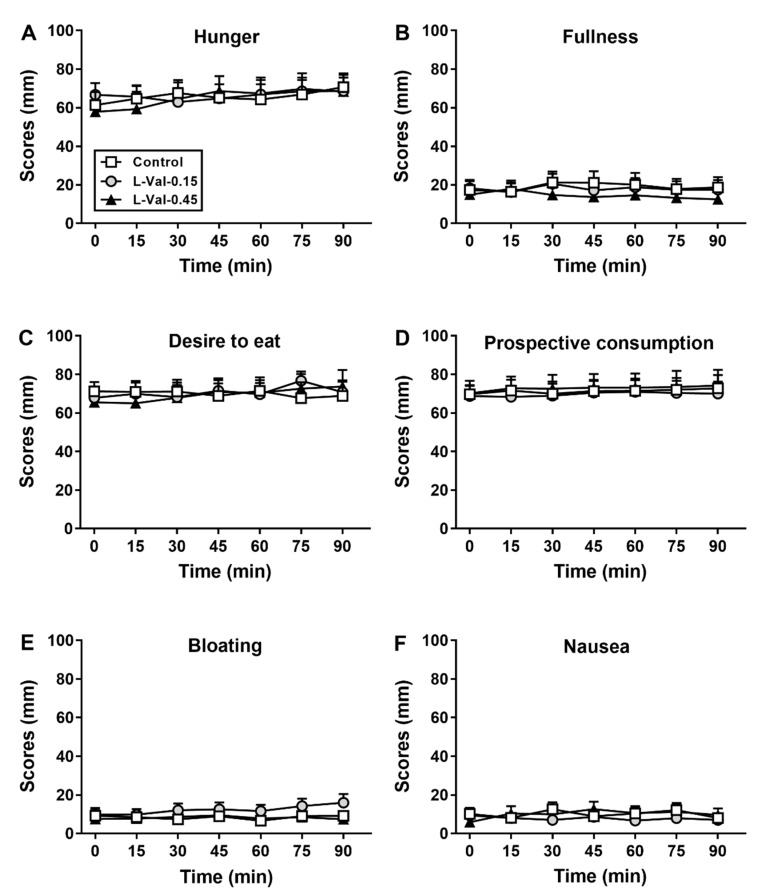
Scores for hunger (**A**), fullness (**B**), desire to eat (**C**), prospective consumption (**D**), bloating (**E**) and nausea (**F**) during 90-min intraduodenal infusions of L-valine at 0.15 kcal/min (“L-Val-0.15”) or 0.45 kcal/min (“L-Val-0.45”), or control. Repeated-measures two-factor ANOVA, with treatment and time as factors, was used to analyse the data. Post-hoc comparisons, adjusted for multiple comparisons by Bonferroni correction, were used to determine significant differences between treatments if ANOVAs were significant. Statistical significance was accepted at *p* < 0.05. Data are means ± SEMs, *n* = 12.

There were no differences in baseline ratings of hunger, fullness, desire to eat, prospective consumption, bloating or nausea between study days. There were also no effects of treatment or time on ratings of these sensations and symptoms (*p* > 0.05 for all; Figure 2A–F).

### 3.3. Antropyloroduodenal Pressures

Baseline values for antral, pyloric and duodenal pressures did not differ between study days and were 0 (*p* > 0.05 for all).

There was no effect of treatment on the total number, mean amplitude or MI of antral PWs; mean basal pyloric pressures; total number or mean amplitude of IPPWs, or the total number, mean amplitude or MI of duodenal PWs (*p* > 0.05 for all; Table 1).

### 3.4. Plasma CCK and Blood Glucose Concentrations

There were no differences in baseline values between study days for plasma CCK or blood glucose concentrations (*p* > 0.05 for all).

**Figure 3:**
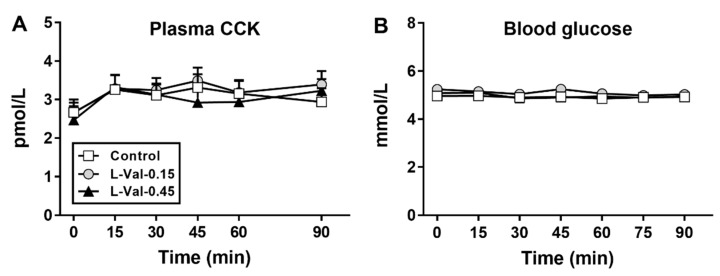
Plasma CCK (**A**) and blood glucose (**B**) concentrations during 90-min intraduodenal infusions of L-valine at 0.15 kcal/min (“L-Val-0.15”) or 0.45 kcal/min (“L-Val-0.45”), or control. Repeated-measures two-factor ANOVA, with treatment and time as factors, was used to analyse the data. Post-hoc comparisons, adjusted for multiple comparisons by Bonferroni correction, were used to determine significant differences between treatments if ANOVAs were significant. Statistical significance was accepted at *p* < 0.05. Data are means ± SEMs, *n* = 11 for CCK (due to technical difficulties with blood sampling), *n* = 12 for glucose.

There was no effect of treatment (*p* > 0.05), but an effect of time (*p* < 0.05), on plasma CCK concentrations (Figure 3A). All 3 infusions slightly increased plasma CCK at *t* = 15 min (*p* < 0.05), after which time no further changes occurred.

There was no effect of treatment, or time, on blood glucose concentrations (*p* > 0.05; Figure 3B).

## 4. Discussion

We have investigated the effects of the BCAA, L-valine, which is abundant in whey protein, to elucidate whether L-valine, like L-leucine and L-isoleucine, has potent effects to reduce energy intake and/or blood glucose and to modulate key underlying GI functions. Our study indicates that L-valine, when administered intraduodenally at loads of 0.15 kcal/min or 0.45 kcal/min for 90 min, has no effect on antropyloroduodenal motility, plasma CCK, energy intake or blood glucose, suggesting that L-valine, unlike L-leucine at these loads, does not have GI, energy intake-suppressant or glucoregulatory effects.

There has been substantial interest in BCAAs, particularly L-leucine and L-isoleucine, in relation to their potential role in mediating the effects of protein, particularly whey, on energy intake, blood glucose and other metabolic outcomes [4,22]. We have reported recently that intraduodenal infusion of L-leucine at the load of 0.45 kcal/min, but not 0.15 kcal/min (loads identical to those used in the current study), significantly reduced subsequent energy intake, associated with stimulation of plasma CCK concentrations, in healthy males [15]. We have also reported that, in response to intraduodenal whey protein, subsequent energy intake is correlated inversely with circulating concentrations of L-leucine, L-isoleucine and L-valine [14]. Based on these findings, we hypothesised that intraduodenal infusion of L-valine would also reduce energy intake. In contrast to our expectation, L-valine was ineffective—it did not affect subsequent energy intake, nor GI functions of relevance to the regulation of energy intake, specifically the stimulation of plasma CCK and pyloric pressures [32,33]. While it is possible that L-valine may be an amino acid that does not play a role in appetite regulation, it is worth noting the important contributory roles of orosensory and gastric influences (which we bypassed in the current study) with gut hormone release and the modulation of energy intake [34,35]. In support, while oral administration of L-phenylalanine has been shown to reduce subsequent energy intake in healthy humans [36], we reported recently that intraduodenal L-phenylalanine was ineffective [37]. Thus, the effects of orally administered L-valine, and, if effective, potential underlying mechanisms, warrant further investigation.

Studies in healthy individuals have established the capacity of both L-leucine and L-isoleucine to reduce elevated blood glucose levels. For example, intragastric administration of 10 g L-leucine or 10 g L-isoleucine, 15 min before consumption of a carbohydrate-containing mixed-nutrient drink, reduced the blood glucose response to the drink by ~1.1 mmol/L [19]. Furthermore, intraduodenal infusion of leucine at 0.45 kcal/min (9.9 g over 90 min), slightly stimulated insulin and reduced fasting blood glucose modestly [15]. In contrast to L-leucine and L-isoleucine, the few studies on the effects of valine on blood glucose have yielded inconsistent findings [22]. In fasted rats, L-valine (0.3 g/kg body weight, equivalent to ~22.5 g in a 75 kg human), given orally 30 min before an oral glucose tolerance test, increased plasma glucose levels, 60 min later by ~20 mg/dl [38], and, at a dose of 1 g/kg body weight, ameliorated the fall in blood glucose induced by exercise [39], while, in contrast, intrahypothalamic infusion of L-valine (12 nmol), given with a continuous intravenous glucose infusion, was reported to lower blood glucose [40], compared with control rats. In fasted healthy humans, intravenous administration of 30 g L-valine resulted in a small stimulation of insulin secretion and decrease in blood glucose concentrations [21]. The L-valine loads used in these studies were extremely high. Moreover, the discrepant effects may be due to species differences (rats vs. humans), as well as the different routes of administration (oral vs. intravenous vs. intrahypothalamic). Our data, using doses spanning physiological and supraphysiological ranges, found no effects on blood glucose concentrations. It should be appreciated that our study design did not include a carbohydrate-containing meal or oral administration of L-valine, and, thus, did not evaluate effects on postprandial blood glucose. This also warrants evaluation.

Some limitations of our study should be recognised. We did not measure all of the key gut and pancreatic hormones involved in energy intake or blood glucose regulation, including ghrelin, peptide tyrosine tyrosine, GLP-1, GIP, insulin and glucagon, because of the lack of effect of L-valine on energy intake, blood glucose and plasma CCK concentrations. Intraduodenal infusion of L-valine was used to exclude influences of orosensory factors and to standardise nutrient delivery to the small intestine, bypassing any inter-individual variations in gastric emptying. Thus, we cannot exclude the possibility that L-valine affects energy intake and/or blood glucose when ingested orally or administered intragastrically, thus, activating multiple afferent pathways concurrently [41,42]. We administered L-valine in isolation, thus, the apparent discrepancy in our findings, i.e., a lack of effect of intraduodenally administered L-valine on energy intake, versus the inverse correlation between plasma L-valine concentrations and energy intake after intraduodenal whey protein infusion in our previous study [14], may indicate that L-valine requires the presence of other amino acids to exert an effect on energy intake and/or blood glucose; this hypothesis may also warrant investigation. Finally, we only studied healthy males, because they have been reported to be most sensitive to dietary manipulations [43], hence, we cannot generalise our findings to outcomes in women, overweight/obese or age, however, we believe a different outcome would be unlikely.

In conclusion, intraduodenal infusions of L-valine, at loads that are moderately (3.3 g) or substantially (9.9 g) above World Health Organization requirement recommendations, do not affect antropyloroduodenal motility, plasma CCK or fasting blood glucose concentrations, or energy intake. These observations indicate that, unlike L-leucine or L-isoleucine, L-valine, does not play a critical role in the regulation of energy intake and/or blood glucose control, providing evidence that different amino acids, even though of similar chemical structure, can have distinct physiological effects.

## Figures and Tables

**Table 1:** Number, amplitude and motility indices of antral and duodenal pressure waves, basal pyloric pressure, and number and amplitude of isolated pyloric pressures waves during 90-min intraduodenal infusions of L-valine at 0.15 kcal/min (“L-Val-0.15”), L-valine at 0.45 kcal/min (“L-Val-0.45”), or control.

	Control	L-Val-0.15	L-Val-0.45	*p* Value
Antral pressure waves				
*Number*	51 ± 18	46 ± 11	35 ± 11	>0.05
*Amplitude, mmHg*	37 ± 8	46 ± 8	34 ± 7	>0.05
*Motility index, mmHg × min*	9 ± 1	10 ± 1	9 ± 1	>0.05
Basal pyloric pressure, mmHg	−0.3 ± 0.9	0.2 ± 1.0	0.4 ± 0.4	>0.05
Isolated pyloric pressure waves				
*Number*	14 ± 5	21 ± 9	11 ± 4	>0.05
*Amplitude, mmHg*	9 ± 3	16 ± 4	14± 4	>0.05
Duodenal pressure waves				
*Number*	445 ± 66	495 ±77	361 ± 65	>0.05
*Amplitude, mmHg*	29 ± 2	29 ± 3	27 ± 2	>0.05
*Motility index, mmHg × min*	15 ± 0.4	15 ± 0.4	14 ± 0.5	>0.05

One-factor ANOVA was used to test for differences in number, amplitude and motility indices of antral and duodenal pressure waves, basal pyloric pressure, and number and amplitude of isolated pyloric pressures waves. Post-hoc comparisons, adjusted for multiple comparisons by Bonferroni’s correction, were used to determine significant differences between treatments if ANOVAs were significant. Data are means ± SEMs, *n* = 12.
